# CLIP-Based Adaptive Graph Attention Network for Large-Scale Unsupervised Multi-Modal Hashing Retrieval

**DOI:** 10.3390/s23073439

**Published:** 2023-03-24

**Authors:** Yewen Li, Mingyuan Ge, Mingyong Li, Tiansong Li, Sen Xiang

**Affiliations:** 1 School of Computer and Information Science, Chongqing Normal University, Chongqing 401331, China; 2021210516048@stu.cqnu.edu.cn (Y.L.); tiansongli@cqnu.edu.cn (T.L.); 2 School of Information Science and Engineering, Wuhan University of Science and Technology, Wuhan 430081, China

**Keywords:** multi-modal retrieval, unsupervised learning, deep hashing, graph convolutional networks, attention mechanism

## Abstract

With the proliferation of multi-modal data generated by various sensors, unsupervised multi-modal hashing retrieval has been extensively studied due to its advantages in storage, retrieval efficiency, and label independence. However, there are still two obstacles to existing unsupervised methods: (1) As existing methods cannot fully capture the complementary and co-occurrence information of multi-modal data, existing methods suffer from inaccurate similarity measures. (2) Existing methods suffer from unbalanced multi-modal learning and data semantic structure being corrupted in the process of hash codes binarization. To address these obstacles, we devise an effective CLIP-based Adaptive Graph Attention Network (CAGAN) for large-scale unsupervised multi-modal hashing retrieval. Firstly, we use the multi-modal model CLIP to extract fine-grained semantic features, mine similar information from different perspectives of multi-modal data and perform similarity fusion and enhancement. In addition, this paper proposes an adaptive graph attention network to assist the learning of hash codes, which uses an attention mechanism to learn adaptive graph similarity across modalities. It further aggregates the intrinsic neighborhood information of neighboring data nodes through a graph convolutional network to generate more discriminative hash codes. Finally, this paper employs an iterative approximate optimization strategy to mitigate the information loss in the binarization process. Extensive experiments on three benchmark datasets demonstrate that the proposed method significantly outperforms several representative hashing methods in unsupervised multi-modal retrieval tasks.

## 1. Introduction

With advances in sensing technology and the proliferation of multi-modal data from different sources (e.g., images, voice and video), it is essential to analyze and process these cross-modal data. People are no longer content with a single form of access to data, which makes it urgent to retrieve multimedia data swiftly and efficiently. However, multi-modal data are massive, heterogeneous and highly dimensional, and the retrieval of multi-modal data takes a great deal of time and storage space [1]. Therefore, it is crucial to reduce the storage space of multi-modal data and improve retrieval performance. Among many retrieval methods, hash retrieval [2,3] has attracted extensive research for its efficient storage and retrieval efficiency.

**Figure 1:**
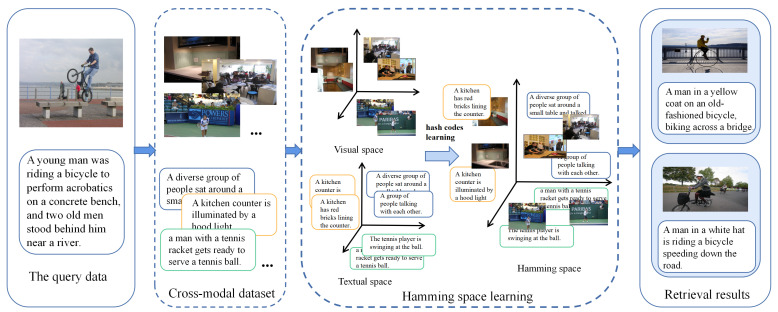
A brief illustration of multi-modal hashing retrieval. The cross-modal hashing method maps the original data into a unified Hamming (binary code) space. Meanwhile, the semantic similarity of the data is preserved during the mapping process. (The more semantically similar the original multimedia data is mapped to a common Hamming space, the closer the distance between their hash codes and vice versa.)

The basic idea of cross-modal hash retrieval [4,5] is to use the sample pair information of different modalities, learn the hash transform of different modalities and map the data of different modalities to a Hamming binary space [6] while keeping the similarity of the data in the process of mapping. (Data with more similar original semantics are projected into the Hamming common space, and the distance between their hash codes is closer.) Fast cross-modal retrieval is then achieved in the Hamming space. Figure 1 illustrates the cross-modal hash retrieval task. Cross-modal hashing can be classified into two categories: supervised and unsupervised methods. Supervised methods [7,8,9] use semantic labels to bridge heterogeneity gaps and semantic gaps, which often achieve better retrieval accuracy. Jiang et al. first proposed a novel approach called deep cross-modal hashing (DCMH) [7], which integrates feature learning and hash code learning into an end-to-end learning framework. However, the large amount of manually annotated label information is expensive, noisy, and difficult to implement in practical scenarios. The unsupervised methods [10,11,12,13] eliminate the dependence on label information and only consider paired multimedia data. Due to the lack of jointly trained label information, unsupervised cross-modal hashing methods suffer from inaccurate training objectives and limited retrieval accuracy. Unsupervised methods have been little explored relative to supervised methods, and this work aims to enhance the retrieval performance of cross-modal hashing under unsupervised conditions.

In recent years, due to the powerful feature extraction capabilities of deep neural networks [14,15,16], unsupervised cross-modal hash retrieval methods based on deep learning have made great progress. The deep unsupervised cross-modal hashing methods [11,17,18] use the deep neural network to extract the feature representation of different modal data and establish the semantic association of different modalities at a high level, thereby achieving large performance improvement. Liu et al. proposed a Joint Modal Distribution-based Similarity Hash (JDSH) [11], which fully preserves the semantic relevance between data by constructing a joint modal similarity matrix and designing a similarity weighting scheme.

Although these unsupervised methods achieve impressive performance, most of them suffer from inaccurate similarity and modal imbalance, leading to sub-optimal retrieval performance. In particular, it is difficult to comprehensively measure complex data correlations with simple data features of different modalities. The original structure of the hash code is destroyed during the process from the real value to the binarization, and there is information loss. In addition, multi-modal learning suffers from imbalance problems due to the modality gap and data bias [19,20], and the training efficiency is still limited.

**Figure 2:**
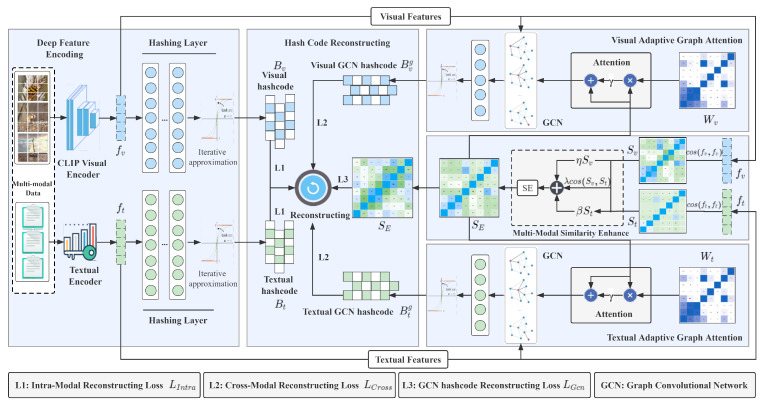
Illustration of our proposed CLIP-based Adaptive Graph Attention Network (CAGAN), which consist of four modules: the deep feature encoding, multi-modal similarity enhancing, adaptive graph attention, and hash codes reconstructing module.

To address these issues, we propose a novel CLIP-based Adaptive Graph Attention Network (CAGAN) for large-scale unsupervised multi-modal hashing retrieval. The framework is shown in Figure 2, and the contributions are as follows:

- We propose a novel unsupervised cross-modal hashing method that uses the multi-modal model CLIP (Contrastive Language-Image Pre-Training) [21,22] to extract cross-modal features and designs a cross-modal similarity enhancement module to integrate the similar information of different modalities, thereby providing a better supervisory signal for hash code learning.
- To alleviate the problem of unbalanced multi-modal learning, an adaptive graph attention module was designed to act as an auxiliary network to assist in the learning of hash functions. The module employs an attention mechanism to enhance similar information and suppress irrelevant information and mines graph neighborhood correlations through graph convolutional neural networks.
- In addition, an iterative approximation optimization strategy is used to reduce the information loss in the hash code binarization process. Sufficient experiments on three benchmark datasets show the proposed method outperforms other state-of-the-art deep unsupervised cross-modal hashing methods.

## 2. Related Work

We briefly introduce related multi-modal hashing work in this section, including deep unsupervised multi-modal hashing, attention-based hashing, and graph-based methods.

### 2.1. Deep Unsupervised Multi-Modal Hashing

Cross-modal hashing methods fall into two categories: supervised methods for labeled multi-modal data and unsupervised methods for paired multi-media data. Unsupervised hashing methods have more research value and application prospects because of their label independence. Deep neural networks [23,24] have shown remarkable ability in encoding deep features of different modal data; thus, deep unsupervised cross-modal hashing retrieval has attracted increasing research. One of the most representative works is deep joint-semantics reconstructing hashing (DJSRH) [10], which proposes a novel method for reconstructing multi-modal hash matrices by designing a joint semantic affinity matrix to unify the similarity relations of different modal data. High-order nonlocal hashing (HNH) [25] constructs a more comprehensive similarity matrix by considering the similarity relationship between multi-modal data from both local and non-local perspectives. DGCPN proposed by Yu et al. [17] preserves the consistency of graph neighbors by integrating information between data and their neighbors and moderates the combined similarity retention loss using three different forms of data similarity. Deep adaptively enhanced hashing (DAEH) [26] proposes a strategy with discriminative similarity guidance and adaptive enhancement optimization that uses information theory to discover weaker hash functions and augment them with additional teacher networks. However, these unsupervised methods suffer from the problem of inaccurate similarity measures, which leads to limited retrieval performance. Inspired by vision and language pre-training (VLP) and related works [21,27,28], we extract cross-modal features using contrastive language–image pre-training (CLIP), which uses the Transformer [16] to achieve fine-grained semantic alignment of image patches and text words and employs contrast learning for large-scale data training. Furthermore, a multi-modal similarity enhancement module is designed to fuse and enhance the similarity information of different modal data, which can effectively alleviate the inaccurate similarity measure of multi-modal data.

### 2.2. Attention-Based Methods

Recently, attention mechanisms [16,29] have attracted extensive attention due to their satisfactory performance in various domains, such as machine translation and image processing. By focusing on the information that is more critical to the current target among the many inputs and reducing the attention to other information or even filtering out irrelevant information, the attention mechanism can solve the information redundancy problem and improve the efficiency and accuracy of the task processing. In recent years, attention-based cross-modal retrieval methods [30,31,32] have been initially explored. Attention-aware deep adversarial hashing (ADAH) [33] proposes an adversarial hash network with an attention mechanism to enhance the measure of content similarity by selectively paying attention to the informative parts of multi-modal data. The self-constraining and attention-based hashing network (SCAHN) [29] proposes an approach for bit-scalable cross-modal hashing that incorporates early and late label constraints into both hash encoding learning and hash representations. Attention-guided semantic hashing (AGSH) [30] adopts an attention mechanism that pays attention to the associated feature features. It can preserve the semantic information in different modal features through the attention module so as to construct an attention-aware semantic affinity matrix. However, unsupervised cross-modal hash retrieval based on an attention mechanism is rarely explored.

### 2.3. Graph-Based Methods

Graph Convolutional Networks (GCNs) [34] have shown excellent performance in learning representations of graph-structured data and have generated extensive research interest in areas such as intelligent transportation, social networks and pharmaceutical medicine. Graph neural networks [34,35] utilize a regression neighborhood aggregation strategy to compute the features of each data node. In recent years, cross-modal hashing methods based on GCNs have received extensive attention [36,37]. In particular, graph convolutional network hashing (GCNH) [38] introduces an asymmetric graph convolution layer that addresses the problems of scalability and out-of-sample extension when exploiting affinity graphs for hashing. Graph convolutional multi-modal hashing (GCMH) [39] proposes multiple modality-individual GCNs under semantic guidance to act on each modality independently to preserve intra-modality similarity, and then fuse the output representations into a fusion graph with an adaptive weighting scheme. Aggregation-based graph convolutional hashing (AGCH) [36] designed an elegant aggregation strategy that leverages multiple similarity measures to build an accurate semantic similarity matrix and employs graph convolutional neural networks to aggregate similarity information across modal data, which further mines the semantic relevance of different modal data. However, these methods cannot comprehensively utilize the features of different modalities to build semantic affinity graphs, resulting in inaccurate relationships between data nodes. Hence, an adaptive graph attention module is designed to address this problem. It uses an attention mechanism to learn a semantic affinity graph and aggregates information between similar nodes through graph convolution, thereby enabling similar data to generate more consistent hash codes. In addition, using GCNs to assist a hash network’s learning can effectively alleviate the problem of multi-modal learning imbalance [40].

## 3. Methodology

In this section, we will elaborate on the proposed CAGAN model, including the following subsections: problem definition and notation, an overview of the model framework, objective function and optimization of the network. It is worth noting that our approach uses batch training and the variables will be represented in a batch manner.

### 3.1. Notation and Problem Definition

To better understand the cross-modal retrieval task and the proposed method, we first introduce some notational definitions used in this paper. Given a cross-modal dataset $O={\left\{v_{i},t_{i}\right\}}_{i=1}^{n}$, where $v_{i}$ and $t_{i}$ represent pairs of image–text, we divide the data into mini-batches of training samples $o=\left\{o_{1},o_{2},\cdots ,o_{j}\right\}$. For each randomly sampled batch of training samples ${\left\{o_{k}=\left[v_{k},t_{k}\right]\right\}}_{k=1}^{m}$, where *m* denotes the batch size, we use $F_{v}\in R^{m\times 512}$ and $F_{t}\in R^{m\times d_{t}}$ for the visual and textual representations. Meanwhile, we denote the hash codes generated by the hash coding network as $B_{v}\in {\left\{-1,+1\right\}}^{m\times c}$ and $B_{t}\in {\left\{-1,+1\right\}}^{m\times c}$, the hash codes generated by the graph convolutional neural network as $B_{v}^{g}\in {\left\{-1,+1\right\}}^{m\times c}$ and $B_{t}^{g}\in {\left\{-1,+1\right\}}^{m\times c}$, where *c* represents the length of the hash code.

In the phase of building the similarity matrix, we first normalize $F_{v}$ and $F_{t}$ to ${\hat{F}}_{v}$ and ${\hat{F}}_{t}$; then, we use the cosine similarity to calculate the visual and textual modality similarity matrices $S_{v}=\cos\left({\hat{F}}_{v},{\hat{F}}_{v}\right)$ and $S_{t}=\cos\left({\hat{F}}_{t},{\hat{F}}_{t}\right)$, respectively, which in turn are used to describe the original image and the inherent similarity between textual data. Furthermore, we can consider the generated hash codes $B_{v}$ and $B_{t}$ as feature vectors that can only contain the vertices of a high-dimensional space. From this perspective, neighboring vertices correspond to similar hash codes; that is, the Hamming distance between two hash codes can be expressed in terms of their cosine angular distance, and the cosine distance of vectors $\vec{x}$ and $\vec{y}$ is defined as follows:

(1) $$\cos\left(x,y\right)=\frac{{\left(\vec{x}\right)}^{T}\vec{y}}{\left|\right|\vec{x}|{|}_{2}\left|\right|\vec{y}|{|}_{2}}\in {\left[-1,+1\right]}^{m\times m}.$$

where ${∥\cdot ∥}_{2}$ denotes the $l_{2}$-normalization of the vectors, and the cosine matrix of the samples reflects the cosine similarity relation between the hash codes, which is equivalent to their Hamming distance relation discussed below. The Hamming distance can be computed as the dot product of two binary codes; it is the number of different characters in a string of equal length and is used to measure the distance between hash codes. Given two hash codes $\;h_{i}$ and $\;h_{j}$, the Hamming distance formula is as follows:

(2) $$dis_{H}\left(h_{i},h_{j}\right)=\frac{1}{2}\left(c-h_{i}⊙h_{j}\right),s.t.h_{i},h_{j}\in {\left\{-1,+1\right\}}^{1\times c}.$$

where *c* is the length of the hash code, and $h_{i}⊙h_{j}$ is the dot product of the hash codes $\;h_{i}$ and $\;h_{j}$. Cross-modal hashing improves retrieval speed and reduces storage consumption by projecting different modal data into a unified Hamming space. It is important to note that the original semantic similarity of the data is preserved in the data projection.

### 3.2. Framework Overview

As depicted in Figure 2, the CAGAN framework is an end-to-end model, which includes four main modules, i.e., the deep feature encoding module, the multi-perspective similarity aggregation module, the adaptive graph attention module, and the hash code reconstruction module. We will elaborate on the implementation process of each module below.

**Deep feature encoding module**. The deep encoding module contains two main networks: visual encoding network and text encoding network. The CLIP-represented vision–language pre-trained (VLP) model has proven to be more effective at learning both textual and visual representations. In this paper, we adopt the CLIP encoder and multi-layer perceptrons (MLPs) as the backbone network, which can extract richer cross-modal semantic features. We denote the visual encoder as $Enc_{v}$ and the textual feature encoder as $Enc_{t}$, while the symbols are expressed as follows:

(3) $$F_{v}=Enc_{v}\left(V,\theta_{v}\right)\in R^{m\times 512},F_{t}=Enc_{t}\left(T,\theta_{t}\right)\in R^{m\times d_{t}}.$$

where *V* and *T* represent batches of image and text training samples. $\theta_{v}$ and $\theta_{t}$ represent the parameters of the visual and textual feature encoding network. Then, we use MLP to learn the hash function; the formula is as follows:

(4) $$H_{v}=MLP_{v}\left(F_{v},\theta_{Hv}\right)\in {\left[-1,+1\right]}^{m\times c},H_{t}=MLP_{t}\left(F_{t},\theta_{Ht}\right)\in {\left[-1,+1\right]}^{m\times c}.$$

Therefore, we can encode the rich semantic features of different modalities to better describe the semantic similarity between the original data and further guide the learning of hash codes.

(5) $$B_{v}=\tanh\left(\alpha H_{v}\right)\in {\left[-1,+1\right]}^{m\times c},B_{t}=\tanh\left(\alpha H_{t}\right)\in {\left[-1,+1\right]}^{m\times c}.$$

where α denotes the number of iterations. As the number of iterations increases, the hyperbolic tangent function converges to a symbolic function: $\lim_{\alpha \to \infty }\tanh\left(\alpha x\right)=sign\left(x\right)$. The iterative approximate optimization strategy is used to mitigate information loss in the hash code binarization process.

**Multi-modal similarity Enhance Module**. Unsupervised hashing methods cannot construct a multi-label similarity matrix to guide the learning of hash codes due to the inability to obtain the labels of the samples. As described in [31,38,41], building a similarity matrix using deep neural networks to capture the complementary and coexistence information of the original data is a superior method, which can provide effective self-supervision for the learning of hash functions. In particular, we use mini-batch visual features $F_{v}={\left\{f_{i}^{v}\right\}}_{i=1}^{m}\in R^{m\times d_{v}}$ to build the visual modality similarity matrix $S_{v}={\left\{s_{ij}^{v}\right\}}_{i,j=1}^{m}\in {\left[-1,+1\right]}^{m\times m}$, where $s_{ij}^{v}=cos\left(f_{i}^{v},f_{j}^{v}\right)$. For the textual modality, we directly leverage the features $F_{t}={\left\{f_{i}^{t}\right\}}_{i=1}^{m}\in R^{m\times d_{t}}$ processed by the bag of words to create the text cosine similarity matrix $S_{t}={\left\{s_{ij}^{t}\right\}}_{i,j=1}^{m}\in {\left[-1,+1\right]}^{m\times m}$, where $s_{ij}^{t}=cos\left(f_{i}^{t},f_{j}^{t}\right)$. Subsequently, we construct a cross-modal similarity matrix to capture the co-occurrence similarity of different modal instances. In particular, we use the visual modality similarity matrix $S_{v}$ and textual modality similarity matrix $S_{t}$ to construct a cross-modal cosine similarity matrix $S_{c}$ that can preserve co-occurrence information between image and text modal instances. The equation for the fusion process is described as follows:

(6) $$S_{c}={\left\{\frac{{\left(s_{i*}^{v}\right)}^{T}\left(s_{j*}^{t}\right)}{\left|\right|s_{i*}^{v}|{|}_{2}\left|\right|s_{j*}^{t}|{|}_{2}}\right\}}_{i,j=1}^{m}=\frac{{\left(S_{v}\right)}^{T}S_{t}}{\left|\right|S_{v}{\left|\right|}_{2}\left|\right|S_{t}{\left|\right|}_{2}}=\cos\left(S_{v},S_{t}\right)\in {\left[-1,+1\right]}^{m\times m}.$$

where ${\left(\cdot \right)}^{T}$ indicates the transposition of the matrix. In addition, we construct a semantically preserved affinity matrix $S_{A}$ that integrates information from different matrices; the formula is expressed as follows:

(7) $$\begin{array}{cc} \; & S_{A}=\eta S_{v}+\beta S_{t}+\lambda S_{c}\in {\left[-1,+1\right]}^{m\times m},\\ \; & s.t.\eta ,\beta ,\lambda \geq 0,\eta +\beta +\lambda =1. \end{array}$$

where η, β, λ are balancing hyper-parameters that trade off the degree of importance of similarity matrices between image and text modalities. Finally, we performed similarity enhancement on the fused affinity matrix $S_{A}$ with the following formula:

(8) $$\begin{array}{c} E^{+}=e^{\left(\frac{s_{ij}-s_{mean}}{s_{\max}-s_{mean}}\right)},E^{-}=e^{\left(-\frac{1}{2}\times \frac{s_{mean}-s_{ij}}{s_{mean}-s_{\min}}\right)} \end{array}$$

where $s_{\max},s_{\min},s_{mean}$ denote the maximum, minimum and mean of the similarity matrix, respectively. The formula for the similarity matrix enhancement is as follows:

(9) $$s_{ij}=\left\{\begin{array}{c} E^{+}s_{ij},\\ E^{-}s_{ij}, \end{array}\right.\begin{array}{c} ifs_{ij}>s_{mean},\\ ifs_{ij}\leq s_{mean}. \end{array}$$

After the similarity enhancement, the similarity enhanced matrix can be formed as: $S_{E}={\left\{s_{ij}\right\}}_{i,j=1}^{m}$. Compared with previous unsupervised methods, this similarity enhancement brings similar data closer and dissimilar data further by setting a threshold, thus providing a better supervision signal for the learning of hash codes.

**Adaptive graph attention module**. The module employs an attention mechanism to learn the similarity matrix of adaptive modalities; the formula is as follows:

(10) $$S_{*}^{att}=S_{E}+\gamma W_{*}S_{E},*\in \left\{v,t\right\}.$$

where $W_{v}$ and $W_{t}$ represent the projection matrices of visual and textual modalities, and γ is a trade-off parameter. In our experiments, we found that a two-layer graph convolutional network has the best expressiveness. Because graph convolutional networks use fixed filters for learning, too many layers generally limit the expression of the network [41]. Subsequently, we pass the attention similarity matrix into a two-layer graph convolutional network that aggregates information between similar nodes to generate more consistent hash codes:

(11) $$\begin{array}{cc} \; & Z_{*}^{\left(1\right)}=\sigma_{1}\left({\tilde{D}}^{-1/2}S_{*}^{att}{\tilde{D}}^{-1/2}F_{*}W^{\left(1\right)}\right),\\ \; & Z_{*}^{\left(2\right)}=\sigma_{2}\left({\tilde{D}}^{-1/2}S_{*}^{att}{\tilde{D}}^{-1/2}Z_{*}^{\left(1\right)}W^{\left(2\right)}\right),*\in \left\{v,t\right\}. \end{array}$$

where $D_{ii}=\sum_{j}s_{ij}$, $W^{\left(1\right)}$ and $W^{\left(2\right)}$ are the matrices of parameters, while $\sigma_{1}$ and $\sigma_{2}$ denote the activation functions for the first and second layers. $Z_{*}^{\left(i\right)}$ represents the output of the *i*-th layer of a visual and textual modality graph convolutional network. Therefore, we can use the attention mechanism to learn the similarity between data. During training, the attention matrix is iteratively updated to maximize the similarity relationship between instances and then aggregate the information of similar nodes through a graph convolutional network to generate a more consistent hash code, which helps improve the image and text retrieval performance. The hash code generated by graph convolution is as follows:

(12) $$B_{v}^{g}=\tanh\left(\alpha Z_{v}^{\left(2\right)}\right)\in {\left[-1,+1\right]}^{m\times c},B_{t}^{g}=\tanh\left(\alpha Z_{t}^{\left(2\right)}\right)\in {\left[-1,+1\right]}^{m\times c}.$$

where α denotes the number of iterations. We used an iterative approximate optimization strategy to optimize the hash code. When $\lim_{\alpha \to \infty }\tanh\left(\alpha x\right)=sign\left(x\right)$, the discrete problem is transformed into a series of continuous optimization problems, which can effectively alleviate the problems of information loss and instability in the process of binarization.

**Hash codes reconstructing module**. We construct the similarity matrices $S_{v}^{B},S_{t}^{B},S_{c}^{B}$, $S_{G}^{Bv},S_{G}^{Bc}$ from the hash codes $B_{v}$, $B_{t}$, $B_{v}^{g}$ and $B_{t}^{g}$ learned by the network, where $S_{*}^{B}=\cos\left(B_{*},B_{*}\right),*\in \left\{v,t\right\}$, $S_{G}^{Bv}=\cos\left(B_{g}^{v},B_{g}^{v}\right)$, $S_{G}^{Bc}=\cos\left(B_{g}^{v},B_{g}^{t}\right)$. Finally, we construct the loss functions with them and the similarity enhancement matrix $S_{E}$. These loss function formulas are as follows:

(13) $$\begin{array}{cc} \; & L_{Intra}=||\mu S_{E}-S_{v}^{B}{\left|\right|}_{F}^{2}+||\mu S_{E}-S_{t}^{B}{\left|\right|}_{F}^{2},\\ \; & L_{Cross}=||\mu S_{E}-S_{c}^{B}{\left|\right|}_{F}^{2}+||\mu S_{E}-{\left(S_{c}^{B}\right)}^{T}{\left|\right|}_{F}^{2}-\frac{1}{m}\sum (S_{v}^{B}⊗S_{t}^{B}),\\ \; & L_{Gcn}=||\mu S_{E}-S_{G}^{Bv}{\left|\right|}_{F}^{2}+||\mu S_{E}-S_{G}^{Bc}{\left|\right|}_{F}^{2}. \end{array}$$

where $L_{Intra}$ and $L_{Cross}$ denote the intra-modal loss and cross-modal loss, respectively. $L_{Gcn}$ represents the graph convolution reconstructing loss. μ is a scale hyper-parameter that can regulate the quantization scope for the enhanced matrix, and the symbol ⊗ indicates the Hadamard matrix product.

### 3.3. Objective Function and Optimization

The parameters of the entire network are iteratively updated through the back-propagation algorithm until the network converges and the reconstructing procedure of the hash codes is finished. The formula of the total loss is as follows:

(14) $$\begin{array}{cc} \; & \min_{B_{v},B_{t}}\;L=\varepsilon L_{Intra}+L_{Cross}+\varphi L_{Gcn},\\ \; & s.t.B_{v},B_{t}\in {\left[-1,+1\right]}^{m\times c}. \end{array}$$

where ε and varphi are trade-off hyper-parameters. Minimizing the above loss function allows similar data to generate more consistent hash codes. The proposed CAGAN method can be iteratively optimized batch by batch. By minimizing the loss in Equation (14), CAGAN assists the learning of the hash network through an adaptive graph attention network, effectively capturing the neighborhood structure and co-occurrence information of the original instance to generate high-quality hash codes. The entire CAGAN model can be optimized using SGD and Adam optimization algorithms, and the process of CAGAN is detailed in Algorithm 1.
**Algorithm 1** CLIP-based Adaptive Graph Attention Hashing**Input:** The training set $O={\left\{v_{k},t_{k}\right\}}_{k=1}^{N}$; the number of iterations: *n*, mini-batch size: *m*, the length of hash codes: *c*, hyper-parameters: α,η,β,λ,ε,φ,γ,μ.**Output:** The parameters of the whole network: the parameters of visual and textual networks $\theta_{v},\theta_{t},\theta_{Hv},\theta_{Ht}$, the parameters of the graph convolutional networks $\theta_{Gv},\theta_{Gt}$.1:Initialize the number of iterations n=02:**repeat**3:    n=n+1;α=√n;4:    Randomly sampling *m* text-image pairs $O_{m}={\left\{v_{k},t_{k}\right\}}_{k=1}^{m}$ to construct mini-batch training data; after that, data enhancement and normalization are performed on the image.5:    Batches of images and texts obtain batches of visual features $F_{v}$ and textual features $F_{t}$ through image and text encoding networks. Calculate the cosine similarity of $F_{v}$ and $F_{t}$ to construct the similarity matrix $\;S_{v},S_{t}$ and affinity matrix $S_{A}$ according to the Equations (6) and (7).6:    The affinity matrix $S_{A}$ is enhanced to obtain the matrix $S_{E}$ according to Equation (8) and (9), then, the matrix $S_{E}$ is used as the input of the graph adaptive attention module to learn the hash code.7:    Forward propagation generate hash codes $B_{v},B_{t},B_{v}^{g},B_{t}^{g}$ to construct hash codes similarity matrices $S_{v}^{B},S_{t}^{B},S_{c}^{Bv},S_{G}^{Bc}$. Afterward, the loss function for each component is constructed according to Equation (13).8:    Compute the loss for the whole network according to the Equation (14).9:    Back propagation through stochastic gradient descent and Adam’s algorithm to optimize the parameters of the network.10:**until** convergence11:**return** the parameters of the entire network $\theta_{*},*\in \left\{v,t,Hv,Ht,Gv,Gt\right\}$.

## 4. Experiments

In this part, to evaluate the effectiveness of the proposed CAGAN, comprehensive experiments were carried out on three multi-media benchmark datasets (MS COCO, MIRFLICKR-25K and NUS-WIDE). Firstly, we briefly introduce the datasets and evaluation metrics. Secondly, the proposed CAGAN was compared with several advanced baseline methods, including LSSH [42], IMH [43], CMFH [44], RFDH [45], UDCMH [6], AGCH [36], UGACH [46], SRCH [37], UKD [47], DJSRH [10], JDSH [11], DSAH [18], HNH [25], DGCPN [17], DUCH [48] and DAEH [26]. For the fairness of the experiments, we put the baseline methods with the same experimental settings together for comparison. Finally, the proposed methods were empirically analyzed by parameter-sensitive analysis, ablation study, training efficiency and visualization.

### 4.1. Datasets

**MIRFLICKR-25K** [49]: The multi-label dataset on the Flickr website currently has 25,000 photos and associated text description labels from 24 different categories. To represent relevant textual content, it also provides a 1386-dimensional feature vector obtained from principal component analysis of binary label vectors. For a fair comparison, we adopted the same setting as the previous methods [26] and randomly selected 5000 and 2000 samples as training and test sets, respectively.

**NUS-WIDE** [50]: The dataset contains 269,648 images collected from real scenes with their corresponding textual descriptions and labels. In this paper, we follow the setup of previous work and select the 10 most widely used concepts and their associated 186,577 image–text pairs. For each text, a 1000-dimensional BOW feature representation was provided by principal component analysis. We randomly selected 2000 samples and 5000 samples as the test and training sets, respectively.

**Figure 3:**
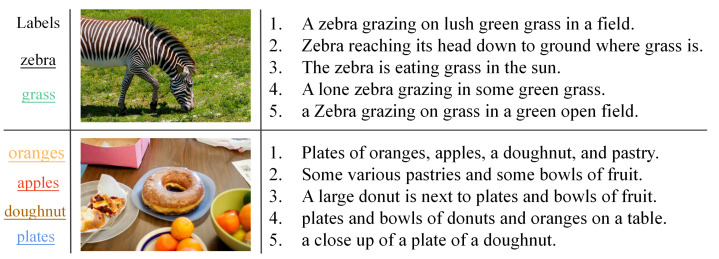
An example of an image from the MS COCO dataset and the captions associated with it. The first column shows the common labels for the images and text, with each image containing five corresponding descriptions.

**MS COCO** [51]: It is a widely used and diverse dataset for object recognition, multimedia retrieval and semantic segmentation. The dataset contains 123,287 images obtained from intricate, everyday scenes where objects in the photographs were localized by careful segmentation. In our experiments, we used 87,081 photographs with 91 categories of information, each corresponding text represented by a 2000-dimensional bag-of-words vector. In addition, we randomly selected 5000 image–text pairs as the query set and the remaining pairs as the retrieval set; 10,000 photo and phrase pairs from the retrieval set were randomly selected as the training set. We show the structure of the MS COCO dataset in Figure 3 and summarize the statistics for the three datasets in Table 1.

### 4.2. Evaluation Metrics

Cross-modal image–text retrieval focuses on two search tasks: “Text-query-Image $\left(T\to I\right)$” and “Image-query-Text $\left(I\to T\right)$”. They use an instance of one modality as a query point to retrieve similar data from another modality in the database. In experiments, we employ two widely used metrics for retrieval measurement: Mean Average Precision (MAP) and the precision of top-N curve to measure the proposed model’s retrieval performance compared with other methods. Precision and ranking information can be reflected well in the measurement methods. In particular, given a query set $Q=[q_{1},q_{2},\cdots ,q_{M}]$, the MAP is denoted as follows:

(15) $$MAP=\frac{\sum_{i=1}^{M}AP(q_{i})}{M},AP=\frac{1}{L_{q}}\sum_{k=1}^{n}P(k)\Delta R\left(k\right).$$

where $q_{i}$ indicates the query instances, and *M* indicates the total number of query instances. In addition, *n* represents the number of instances in the dataset, *k* denotes the number of instances returned during the search procedure and $L_{q}$ indicates the number of data in the dataset associated with the query data, i.e., the total amount of data instances. P(k) is the accuracy rate of the top *k* samples retrieved during the search procedure. ΔR(k) represents the recall value as the number of instances ranging from k−1 to *k*. Average precision is equal to the average retrieval accuracy in a single query datum. The precision of the top-N curve is also an important measurement indicator that indicates the precision at various numbers of retrieved instances. The precision of the top-N curve represents the average accuracy of the top N after the retrieval results are sorted, which reflects the generalization ability and comprehensive performance of a model.

### 4.3. Implementation Details

For experimental environments, the proposed CAGAN method was implemented with the PyTorch platform and 32 GB memory on an NVIDIA RTX 3060 GPU. Using cross-validation, the hyper-parameters were determined as follows: $\left\{\eta =0.5,\beta =0.2,\lambda =0.3\right\}$, σ=0.1,ε=1 and μ=1.4 for all datasets, γ=0.45,φ=0.15 for MIRFLICKR-25K and $\left\{\gamma =0.5,\varphi =0.25\right\}$ for NUS-WIDE and MSCOCO. For the MAP evaluation, the number of ranked samples for MIRFLICKR-25K, NUS-WIDE and MSCOCO was set to 5000.

In the experiments, we use the CLIP (VIT/B-16) [21] model pre-trained on 400 million image–text pairs and the bag-of-words model for the feature encoding module. VIT/B-16 represents one of the models of CLIP, which uses the vision transformer [24] to model image modal data. Subsequently, we construct similarity matrices for different modalities from the encoded image and text features. For the adaptive graph attention module, we perform adaptive attention learning on the similarity-enhanced affinity matrix, which consists of two graph convolutional layers and a multi-layer perceptron $\left(512\to 1024\to C\right)$. Finally, we reconstruct the hash codes generated by the multi-layer perceptron ($D_{f}\to 4096\to C$, where $D_{f}$ represents the dimension of image or text features, and *C* represents the length of the hash code and the graph convolutional network to generate more consistent hash codes for related data through Equations (11) and (12). For the optimization process of the network, we adopt the SGD and Adam optimizer with a learning rate of 0.01, weight decay of 5e-4 and momentum of 0.9. The batch size is set to 32 for three benchmark datasets at the training stage.

### 4.4. Comparison Results and Discussions

In experiments, we compare two cross-modal retrieval tasks: I→T and T→I: using image query texts and vice versa. In this subsection, we compare the retrieval performance of all baselines and CAGAN in terms of MAP and Top-N precision curves in the two retrieval tasks, respectively.

**MAP comparison results**: Table 2 displays the MAP@5000 results of the proposed CAGAN compared with other state-of-the-art unsupervised cross-modal hashing methods at hash code lengths from 16 bits to 128 bits on three benchmark datasets (MIRFlickr-25K, NUS-WIDE and MS COCO). As can be seen from the data in Table 2, our proposed method outperforms all compared baselines. It is worth noting that the first four approaches are traditional methods, and the rest are deep-neural-network-based methods. The methods based on deep neural networks have achieved great performance improvement because of the strong nonlinear feature extraction capabilities of neural networks. Compared to some advanced unsupervised cross-mode hashing baselines, our method has about 1.5–3% performance improvement, which confirms the superiority of the proposed CAGAN. In addition, the improvement in our proposed method on the NUS-WIDE dataset is relatively small—about 0.6–2.2%—because the NUS-WIDE dataset contains a small number of categories. The performance improvement of our method is more obvious on MSCOCO with a large number of categories and still maintains a good performance in the case of a lower hash code length. It reflects the excellent ability of the proposed model for fine-grained retrieval, and it is more suitable for practical application.

To further verify the effectiveness of the proposed CAGAN, we compare five additional deep cross-modal unsupervised hashing methods in the MIRFLICKR-25K and NUS-WIDE datasets, and the comparison results are presented in Table 3 and Table 4. We compare methods with the same experimental setup together in Table 3. It can be seen that our proposed method outperforms all comparison methods in the MAP@50 and MAP@ALL settings. On the MIRFLICKR-25K dataset, the proposed method outperforms the existing methods in MAP@50, even when compared with the state-of-the-art AGCH method. The proposed method has 2–4% performance improvement. On the NUS-WIDE dataset, GAGAN’s MAP@50 has a 1–2% improvement in retrieval accuracy compared with the compared methods. In addition, the MAP@ALL performance on both datasets is also significantly improved. The results in Table 3 further illustrate the effectiveness of the CAGAN method.

**Figure 4:**
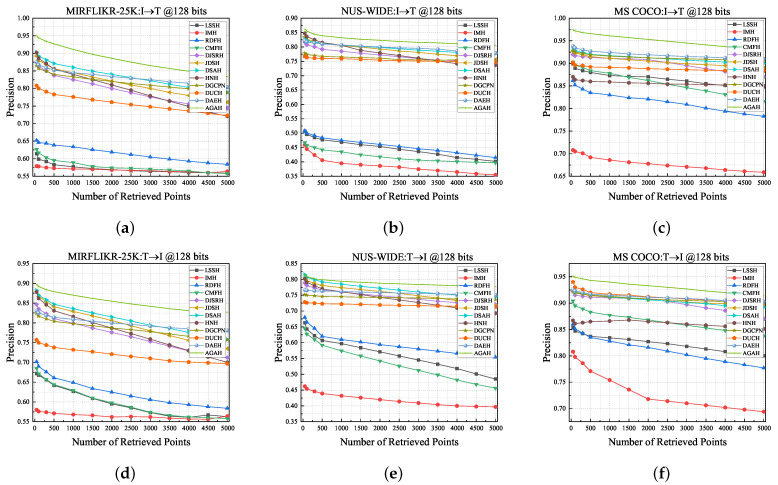
The precision of top-N curves with 128 bits compared to eleven unsupervised methods on three benchmark datasets. (**a**–**c**) show the top-N accuracy curves for image retrieved text (I→T) on MIRFLICKR-25K, NUS-WIDE, and MS COCO datasets, respectively. (**d**–**f**) display the top-N accuracy curves for text retrieved image (T→I) on the three datasets, respectively.

**Top-N precision curves**: Figure 4 shows the top-N precision curves of the proposed method and all eleven baseline methods compared on three multimedia datasets. The top-N accuracy curves are drawn by changing the number of retrieved samples from 1 to 5000, and it reflects the model’s fluctuations in retrieval accuracy as the number of retrievals increases. As can be seen from the curves in Figure 3, our method outperforms all contrasting baselines, which intuitively reflects the efficiency of our CAGAN. It is worth noting that as the number of retrieved instances increases, the top-N precision curve decreases slowly. A reasonable explanation is that our proposed adaptive graph attention module can assist the learning of hash codes, thereby generating more high-quality hash codes. Finally, together with the MAP comparison results, the top-N precision curve can also illustrate that our proposed method mitigates the loss of accuracy in the process of binarization, thus improving retrieval performance and maintaining a high accuracy rate as the number of retrieved samples grows.

### 4.5. Ablation Study

To demonstrate the effectiveness and contribution of each module in our proposed approach, ablation experiments were carried out for each module. To this end, five variants of the model were designed to verify the impact of each module on the overall model. The results of the ablation experiments are shown in Table 5. These variant models are elaborated as follows:

- CAGAN-1: CAGAN-1 indicates that the variant model uses only visual similarity. It uses only the cosine similarity of the image modalities to construct the similarity matrix as a supervised signal.
- CAGAN-2: It indicates that the variant uses only textual similarity, which uses only the cosine similarity of the text modalities to construct the similarity matrix as a supervised signal.
- CAGAN-3: It refers to the variant without the adaptive graph attention module; the adaptive graph attention module can further aggregate information from similar data to produce consistent hash codes.
- CAGAN-4: To alleviate the problem of information loss during binarization, we adopt an iterative approximate optimization strategy to generate hash codes. CAGAN-4 indicates that the variant does not employ an iterative approximate optimization strategy.
- CAGAN-5: We removed the attention mechanism from the model to test whether the attention mechanism could learn similarities between different modal data.

The MAP results for the different variants on three multi-media datasets are presented in Table 4. Accordingly, we can conclude the following:

- Analysis of Table 4 shows that each module plays a significant role in the overall model. Among them, CAGAN-2 has the most obvious performance drop because language is human-refined information, and the similarity matrix constructed from text is sparse. However, CAGAN-1 only uses image features to build a similarity matrix but with less performance degradation. One potential reason for this is that images contain richer, fine-grained semantic information. The results from CAGAN-1 and CAGAN-2 demonstrate the effectiveness of our proposed multi-modal similarity enhancement module.
- The adaptive graph attention module also has an impact on the performance of the proposed CAGAN. Specifically, from the results of CAGAN-3 and CAGAN-5, it can be seen that both the graph convolutional neural network and the attention mechanism contribute to the performance improvement of the model by about 1.5–2.5%.

In addition, we performed ablation experiments on different backbone networks, and the MAP results on MIRFLICKR-25K are shown in Table 6. We find that using CLIP as the backbone network has the best performance, followed by ResNet-152, which reflects that CLIP has excellent visual-linguistic feature extraction ability and is well-suited for cross-modal tasks.

### 4.6. Parameter Sensitivity Analysis

**Figure 5:**
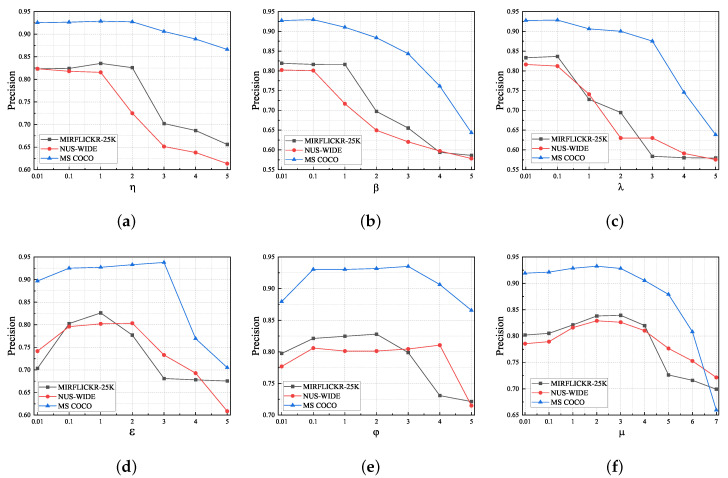
The parameter sensitivity analysis for η,β,λ,ε,φ,μ on three benchmark datasets at 128 bits. (**a**) indicates the change in model precision as the image similarity weights increase. (**b**) represents the change in retrieval precision as the text similarity weights increase. (**c**) λ is a trade-off parameter for cross-modal similarity. (**d**) ε weighs the contribution of intra-modal loss. (**e**) φ weighs the contribution of the auxiliary GCN loss. (**f**) μ is a scale hyper-parameter that can regulate the quantization scope for the similarity matrix.

We analyze several hyper-parameters that could affect the results of the proposed method, and the analysis results are shown in Figure 5. The analysis was carried out by the controlled variable method, where one parameter was changed in the experimental setup and the values of the other parameters were fixed. η and β modulate the effect of image and text similarity on model performance, respectively. It is observed that η and β remain relatively stable around the range of 0.01 to 2, and when they are larger than 2, a large drop occurs. λ is a trade-off parameter for cross-modal similarity; it remains stable around 0.1, and when λ>0.1, there is a significant decline. Therefore, properly adjusting the similarity can lead to satisfactory results. Analysis of the results in Figure 5 shows that our method is not sensitive to the choice of ε and φ in the range [0.1, 2], ε and φ weigh the contribution of intra-modal loss and graph convolution loss, and proper adjustment can make the model achieve optimal performance. μ is a scale hyper-parameter that can regulate the quantization scope for the matrix, which can adjust the matrix value to a reasonable range and improve the retrieval performance. In summary, reasonable tuning of the parameters allows the model to maintain advanced retrieval performance, and the proposed method is robust to hyper-parameters within a reasonable interval.

### 4.7. Training Efficiency and Convergence Testing

**Figure 6:**
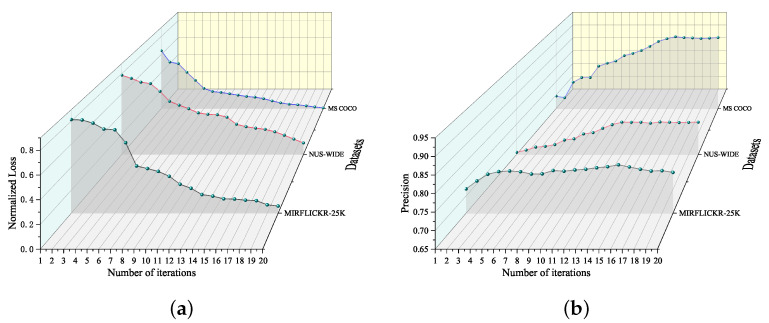
The loss function convergence curves and MAP change curves of the proposed CAGAN on three widely used multimedia datasets at 16-bit code length. (**a**) represents the loss change curve of the model as the number of training iterations increases. (**b**) indicates the retrieval accuracy (MAP@5000) curve of the model as the number of training iterations increases.

In this subsection, we investigate the convergence and training efficiency of the proposed CAGAN on three baseline datasets. Figure 6a shows the final loss function convergence curve at 16-bit hash code length, and Figure 6b displays the change curve of MAP as the number of iterations increases.

The following conclusions can be drawn from the results in Figure 6. First, as the number of optimization iterations increases, the loss function gradually decreases, and the results show that the optimization process can improve the encoding ability of the hash function. In addition, the loss function can converge to the optimal result after dozens of iterations, illustrating that our method reduces training time consumption and improves training efficiency. Finally, the results show that the proposed network converges to the optimal point within dozens of iterations, validating that our proposed network is suitable for unsupervised hash retrieval tasks.

Table 7 shows the computational complexity and training inference time of the proposed method and several advanced models on the MIRFLICKR-25K dataset. Although our proposed method is larger than the other methods in terms of number of parameters, our method converges faster due to our use of a multimodal model with frozen weights. In summary, the proposed CAGAN is more advantageous in terms of both retrieval accuracy and training time.

### 4.8. Visualization

**Figure 7:**
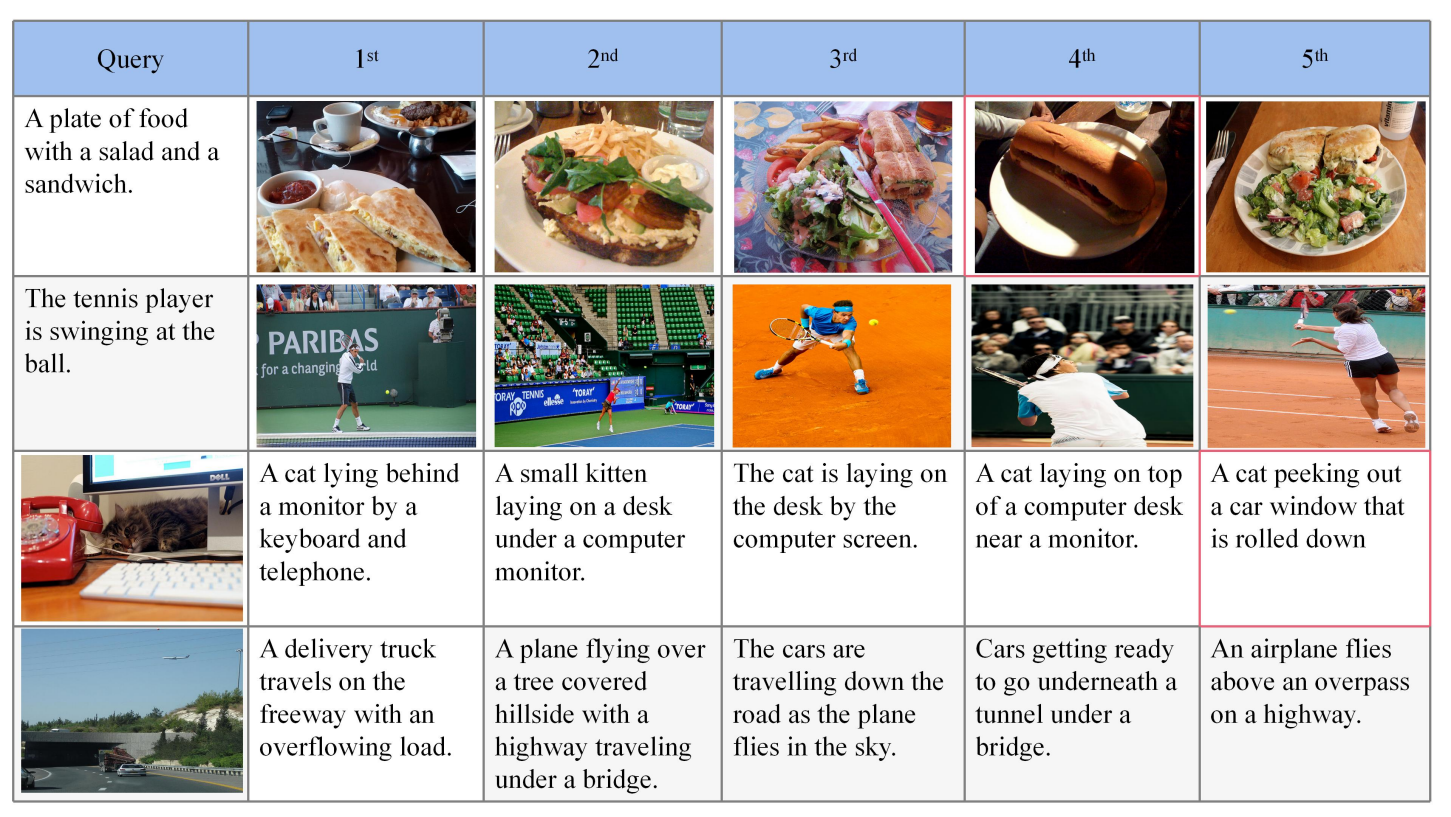
The visualization of cross-modal hash retrieval results. The first column is query data and the rest of the columns are the top five retrieved results. The data in the red box indicate that semantically incompatible data were retrieved.

Figure 7 shows an example of the visualization results of CAGAN on the image and text retrieval task. The first column is the sample queried, the hash code is generated from the queried sample, the Hamming distance is calculated in the database through the hash code and the top five most similar results retrieved after Hamming sorting are displayed in the remaining columns. It is worth noting that the data boxed in red in Figure 7 indicate data that do not quite match the semantics of the query. One potential reason for this is that there is not enough data similar to the query due to data bias. Although these semantically incompatible data are retrieved, they are somehow related to the query data. Overall, it can be observed that the proposed method returns plausible retrieval results through Hamming sorting.

## 5. Conclusions

In this paper, to solve the problem of multi-modal data retrieval generated by different sensors, we proposed an effective and novel CLIP-based Adaptive Graph Attention Network applied to unsupervised multi-modal hashing retrieval tasks. To the best of our knowledge, we first apply CLIP to unsupervised multi-modal hashing. We designed a multi-modal similarity enhancement module to enhance data similarity, which helps improve retrieval accuracy. In addition, an iterative approximation optimization strategy is used to reduce the information loss during hash code binarization. Finally, a well-designed graph adaptive attention module can assist the learning of the hash network and alleviate the problem of unbalanced multi-modal learning. Sufficient experiments carried out on three benchmark datasets demonstrate that the proposed method outperforms several representative advanced methods. In the future, we will further investigate the performance of CAGAN on other retrieval tasks.

## Figures and Tables

**Table 1:** The Statistics of Three Cross-modal Retrieval Benchmark Datasets.

Datasets	MIRFLICKR-25K	NUS-WIDE	MS COCO
Database	25,000	186,577	123,287
Training	5000	5000	10,000
Testing	2000	2000	5000
Labels	24	10	91

**Table 2:** The MAP@5000 results on cross-modal retrieval tasks (I→T indicates the image search text task and vice versa) and three benchmark datasets. The best outcomes are highlighted in bold, and sub-optimal results are underlined.

Task	Method	MIRFLICKR-25K				NUS-WIDE				MS COCO			
		16 bits	32 bits	64 bits	128 bits	16 bits	32 bits	64 bits	128 bits	16 bits	32 bits	64 bits	128 bits
**I→T**	LSSH [42]	0.6756	0.6777	0.6821	0.6844	0.6780	0.7060	0.7029	0.6947	0.8127	0.8320	0.8386	0.8481
	IMH [43]	0.6816	0.6594	0.6434	0.6333	0.6072	0.6237	0.6193	0.5910	0.7372	0.6869	0.6807	0.6591
	RFDH [45]	0.6366	0.6483	0.6587	0.6814	0.5511	0.5724	0.6083	0.6496	0.6902	0.7106	0.7493	0.7826
	CMFH [44]	0.6868	0.6925	0.7011	0.7187	0.6353	0.6641	0.6997	0.7317	0.7252	0.7576	0.7776	0.8162
	DJSRH [10]	0.6729	0.7015	0.7304	0.7443	0.5872	0.6715	0.7177	0.7437	0.7542	0.8156	0.8614	0.8614
	JDSH [11]	0.7254	0.7312	0.7524	0.7615	0.6781	0.7248	0.7434	0.7565	0.6905	0.7584	0.8884	0.8902
	DSAH [18]	0.6395	0.7663	0.7793	0.7898	0.7243	0.7530	0.7720	0.7780	0.8507	0.8813	0.9007	0.9005
	HNH [25]	0.7305	0.7449	0.7385	0.7211	0.6843	0.7215	0.7405	0.7374	0.8305	0.8552	0.8686	0.8502
	DGCPN [17]	0.7599	0.7815	0.7796	0.7880	0.7158	0.7456	0.7559	0.7538	0.8805	0.9020	0.9021	0.9063
	DUCH [48]	0.6670	0.6887	0.7064	0.7230	0.6866	0.7144	0.7282	0.7469	0.8472	0.8666	0.8767	0.8837
	DAEH [26]	0.7826	0.7940	0.8004	0.8047	0.7309	0.7542	0.7728	0.7794	0.8946	0.9029	0.9058	0.9104
	**CAGAN**	**0.7902**	**0.8195**	**0.8248**	**0.8341**	**0.7565**	**0.7890**	**0.7950**	**0.8059**	**0.9166**	**0.9205**	**0.9290**	**0.9322**
**T→I**	LSSH [42]	0.6482	0.6535	0.6623	0.6602	0.5669	0.5878	0.6240	0.6286	0.7085	0.7452	0.7795	0.8003
	IMH [43]	0.6812	0.6673	0.6543	0.6399	0.6260	0.6442	0.6384	0.6175	0.7682	0.7172	0.7152	0.6942
	RFDH [45]	0.6251	0.6460	0.6544	0.6637	0.5512	0.5684	0.5927	0.6304	0.7012	0.7172	0.7413	0.7774
	CMFH [44]	0.6611	0.6699	0.6799	0.6953	0.6092	0.6418	0.6726	0.6966	0.7577	0.7895	0.8099	0.8382
	DJSRH [10]	0.6756	0.6909	0.6985	0.7124	0.6010	0.6567	0.7076	0.7197	0.7593	0.8326	0.8621	0.8697
	JDSH [11]	0.6989	0.7192	0.7241	0.7354	0.6749	0.7155	0.7115	0.7181	0.7581	0.8296	0.8949	0.8952
	DSAH [18]	0.6462	0.7540	0.7593	0.7586	0.6688	0.7167	0.7484	0.7457	0.8546	0.8868	0.8904	0.8919
	HNH [25]	0.7234	0.7204	0.7060	0.7002	0.6711	0.6996	0.6962	0.6931	0.8398	0.8635	0.8669	0.8517
	DGCPN [17]	0.7273	0.7507	0.7571	0.7575	0.7023	0.7230	0.7426	0.7362	0.8807	0.8978	0.8991	0.9015
	DUCH [48]	0.6521	0.6684	0.6818	0.6972	0.6619	0.6943	0.7097	0.7130	0.8607	0.8855	0.8980	0.9032
	DAEH [26]	0.7607	0.7676	0.7743	0.7814	0.7132	0.7335	0.7485	0.7510	0.8882	0.8988	0.9007	0.9033
	**CAGAN**	**0.7790**	**0.8018**	**0.8160**	**0.8272**	**0.7350**	**0.7472**	**0.7676**	**0.7697**	**0.9048**	**0.9057**	**0.9072**	**0.9172**

**Table 3:** The MAP comparison results with extra methods on the MIRFLICKR-25K dataset.

Configurations	Method	I→T				T→I			
		16 bits	32 bits	64 bits	128 bits	16 bits	32 bits	64 bits	128 bits
MAP@50 in [6]	UDCMH [6]	0.689	0.698	0.714	0.717	0.692	0.704	0.718	0.733
	AGCH [36]	0.865	0.887	0.892	0.912	0.829	0.849	0.852	0.880
	**CAGAN**	**0.904**	**0.929**	**0.928**	**0.941**	**0.882**	**0.889**	**0.898**	**0.901**
MAP@All in [46]	UGACH [46]	0.676	0.693	0.702	0.706	0.676	0.692	0.703	0.707
	SRCH [37]	0.680	0.691	0.699	*	0.697	0.708	0.715	*
	UKD [47]	0.700	0.706	0.709	0.707	0.704	0.705	0.714	0.712
	**CAGAN**	**0.708**	**0.723**	**0.714**	**0.725**	**0.715**	**0.722**	**0.731**	**0.743**

**Table 4:** The MAP comparison results with extra methods on the NUS-WIDE dataset.

Configurations	Method	I→T				T→I			
		16 bits	32 bits	64 bits	128 bits	16 bits	32 bits	64 bits	128 bits
MAP@50 in [6]	UDCMH [6]	0.511	0.519	0.524	0.558	0.637	0.653	0.695	0.716
	AGCH [36]	0.809	0.830	0.831	0.852	0.769	0.780	0.798	0.802
	**CAGAN**	**0.816**	**0.827**	**0.845**	**0.862**	**0.782**	**0.791**	**0.804**	**0.815**
MAP@All in [46]	UGACH [46]	0.613	0.623	0.628	0.631	0.603	0.614	0.640	0.641
	SRCH [37]	0.544	0.556	0.567	*	0.553	0.567	0.575	*
	UKD [47]	0.584	0.578	0.586	0.613	0.587	0.599	0.599	0.615
	**CAGAN**	**0.628**	**0.641**	**0.647**	**0.656**	**0.632**	**0.643**	**0.658**	**0.662**

**Table 5:** The MAP@5000 results on image–text retrieval tasks (I→T indicates the image search text task and vice versa) at various code lengths and datasets. The best outcomes are highlighted in bold.

Task	Method	Configuration	MIRFLICKR-25K		NUS-WIDE		MS COCO	
			32 bits	128 bits	32 bits	128 bits	32 bits	128 bits
I→T	CAGAN-1	$S=S_{v}$	0.7660	0.8155	0.7415	0.7954	0.9030	0.9266
	CAGAN-2	$S=S_{t}$	0.7578	0.7975	0.7495	0.7722	0.8643	0.9036
	CAGAN-3	−(φ=0.15)	0.7965	0.8107	0.7776	0.7998	0.8981	0.9137
	CAGAN-4	−(α=1)	0.7960	0.8123	0.7711	0.7910	0.9036	0.9121
	CAGAN-5	−(γ=0.45)	0.7969	0.8225	0.7738	0.7875	0.9045	0.9152
	**CAGAN**	**ALL**	**0.8195**	**0.8341**	**0.7890**	**0.8059**	**0.9205**	**0.9322**
T→I	CAGAN-1	$S=S_{v}$	0.7603	0.8053	0.7339	0.7553	0.8754	0.9098
	CAGAN-2	$S=S_{t}$	0.7561	0.7787	0.7411	0.7551	0.8600	0.8867
	CAGAN-3	−(φ=0.15)	0.7954	0.8041	0.7451	0.7654	0.8537	0.8876
	CAGAN-4	−(α=1)	0.7830	0.8131	0.7531	0.7647	0.8852	0.9053
	CAGAN-5	−(γ=0.45)	0.7757	0.8000	0.7352	0.7550	0.8867	0.9042
	**CAGAN**	**ALL**	**0.8018**	**0.8272**	**0.7472**	**0.7697**	**0.9057**	**0.9172**

**Table 6:** The MAP@5000 of the proposed CAGAN with different backbone networks on the MIRFLICKR-25K dataset.

	MIRFLICKR-25K			
Backbones	I→T		T→I	
	32-bits	128-bits	32-bits	128-bits
AlexNet [15]	0.7763	0.7951	0.7622	0.7894
Densenet [23]	0.8212	0.8313	0.7982	0.8132
ResNet-50 [14]	0.7905	0.8122	0.7774	0.8036
ResNet-152 [14]	0.8230	0.8267	0.8033	0.8274
VIT-B-16 [24]	0.7780	0.8131	0.7714	0.8066
**CLIP-B/16** [21]	**0.8196**	**0.8342**	**0.8014**	**0.8272**

**Table 7:** Computational complexity and time cost (in seconds) of various models on MIRFLICKR-25K.

Method	Parameter	Training Time				Query Time			
		16 bits	32 bits	64 bits	128 bits	16 bits	32 bits	64 bits	128 bits
UDCMH [6]	∼364M	1101.55	1129.36	1157.24	1149.12	58.22	56.48	59.13	59.28
DJSRH [10]	∼368M	995.58	1029.27	1032.88	1025.11	57.51	59.30	59.96	58.47
AGCH [36]	∼385M	1122.18	1124.43	1130.68	1139.86	59.46	58.88	59.29	59.58
Ours	∼393M	**1110.50**	**1115.42**	**1125.78**	**1130.25**	**59.48**	**59.56**	**58.72**	**59.60**

## Data Availability

The data and source code supporting the findings of this study are available in https://github.com/AwakerLee/CAGAN, and accessed on 5 December 2022.
